# Clinical Adoption and Perspectives on Bioceramic and Bioactive Materials Among Dental Specialists: A Cross-Sectional Study

**DOI:** 10.7759/cureus.94308

**Published:** 2025-10-10

**Authors:** Ketaki Rajguru, Charu Khurana, Khushbu Barak, Syed A Haque, Pransi Gupta, Sumit Goyal, Seema Gupta

**Affiliations:** 1 Department of Conservative Dentistry and Endodontics, Tatyasaheb Kore Dental College and Research Centre, Kolhapur, IND; 2 Department of Public Health Dentistry, Faculty of Dental Sciences, Shree Guru Gobind Singh Tricentenary University, Gurugram, IND; 3 Department of Conservative Dentistry and Endodontics, Faculty of Dental Sciences, Shree Guru Gobind Singh Tricentenary University, Gurugram, IND; 4 Department of Pedodontics and Preventive Dentistry, Kothiwal Dental College and Research Centre, Moradabad, IND; 5 Department of Periodontology, Kothiwal Dental College and Research Centre, Moradabad, IND; 6 Department of Public Health Dentistry, Surendera Dental College and Research Centre, Sriganganagar, IND; 7 Department of Orthodontics, Kothiwal Dental College and Research Centre, Moradabad, IND

**Keywords:** bioactive, bioceramic, dentistry, regenerative, survey

## Abstract

Introduction: Bioceramics and bioactive materials have transformed modern dentistry, particularly in regenerative endodontics and minimally invasive procedures, owing to their exceptional biocompatibility and their ability to promote tissue regeneration. Understanding the factors influencing knowledge, attitude, and practice (KAP) toward these materials is crucial for advancing their clinical application. This study explored how sex, specialty, and academic rank shape KAP among dental specialties with the aim of identifying barriers for the integration of bioceramics and bioactive materials in dental education and practice.

Materials and methods: A cross-sectional survey was conducted in the Department of Conservative Dentistry and Endodontics. The study involved 420 participants recruited from 16 dental colleges across India, with four colleges from each geographical zone (North, South, East, and West), using a secure online survey platform. Participants, categorized by sex, specialty (endodontists, pedodontists, public health dentists, prosthodontists), and academic rank (senior lecturer, reader, professor), completed a validated questionnaire assessing KAP toward bioceramics and bioactive materials. The KAP scores were evaluated using median values. Non-parametric tests (Mann-Whitney U and Kruskal-Wallis) were used to assess the differences across variables. Spearman’s correlation was used to examine the relationships between the KAP scores and experience, specialty, and rank. Network analysis was used to analyze the influence of hierarchies within academic ranks and specialties.

Results: A total of 370 (88%) dental specialists responded. Significant differences were observed across groups. Male participants and endodontists exhibited higher knowledge and practice scores, whereas female participants showed more positive attitudes. Professors outperformed other ranks in knowledge and practice, but senior lecturers displayed less favorable attitudes. Experience was strongly correlated with higher knowledge and practice scores but negatively correlated with attitude. Network analysis identified professors and endodontists as key influencers, with senior lecturers and prosthodontists showing a lower influence. Attitudes remained consistently positive across specialties.

Conclusion: Sex, specialty, and academic rank significantly influenced KAP toward bioceramics and bioactive materials, with professors and endodontists leading to better adoption. Sex-neutral, cross-specialty training and targeted interventions for senior lecturers could bridge knowledge gaps and enhance attitudes, promoting wider use of these materials in regenerative dentistry.

## Introduction

In dentistry, restorative and regenerative procedures play a pivotal role in preserving tooth structure, promoting tissue healing, and enhancing long-term clinical outcomes [1]. Restorative dentistry focuses on repairing damaged teeth through fillings, crowns, and other interventions, whereas regenerative approaches aim to restore lost or diseased tissues, such as pulp, dentin, and periodontal structures, by stimulating biological repair mechanisms [1,2]. Over the past few decades, the emergence of bioceramic and bioactive materials has revolutionized these domains, offering superior biocompatibility, bioactivity, and therapeutic potential compared to traditional materials, such as amalgam or resin composites [3,4].

Bioceramic materials, typically derived from calcium silicates, phosphates, or hydroxyapatite, exhibit excellent sealing properties, antibacterial effects, and the ability to induce mineralization. Examples include mineral trioxide aggregate and calcium silicate-based cements, which are widely used in endodontic procedures such as vital pulp therapy, apexification, and root-canal filling [3,5]. Bioactive materials, a broader category encompassing bioceramics, release ions, such as calcium, phosphate, and silica, to interact with surrounding tissues, promoting remineralization and tissue regeneration. These materials are applied in restorative fillings that release fluoride or other ions to prevent secondary caries, as well as in periodontal regeneration for bone grafting and guided tissue regeneration [4,6]. Their bioactivity fosters a dynamic interface with biological systems, reducing inflammation, enhancing adhesion, and supporting stem cell differentiation, which are crucial for regenerative success [7].

Despite these advantages, the clinical adoption of bioceramic and bioactive materials varies across dental specialties and is influenced by factors such as cost, handling complexity, and familiarity with evidence-based protocols [8]. Previous studies have highlighted gaps in knowledge and attitudes toward related concepts, such as regenerative endodontics and biomimetic approaches, particularly among general practitioners and specialists [8,9]. For instance, while endodontists often demonstrate higher awareness due to their frequent use in root canal therapies, prosthodontists and general dentists may underutilize these materials owing to perceived barriers in training or integration into routine practice. This disparity underscores the need for cross-specialty insights to identify educational needs, attitudinal shifts, and facilitators of broader implementation.

This study aims to assess how sex, dental specialty, and academic rank influence the knowledge, attitudes, and practices (KAP) of dental faculty regarding bioceramics and bioactive materials, with the goal of identifying specific barriers and facilitators to their adoption in regenerative and restorative dentistry to inform targeted educational and clinical interventions. Understanding these dynamics is essential for advancing minimally invasive dentistry and aligning clinical practices with biomaterial innovation.

## Materials and methods

Study design and setting

This study was designed as a cross-sectional survey in the Department of Conservative Dentistry and Endodontics, Tatyasaheb Kore Dental College and Research Centre, Kolhapur, India, spanning from April 2025 to July 2025, encompassing questionnaire development in the initial month, validation and pilot testing in May, data collection over June and early July, and preliminary analysis by the end of July. Ethical approval was obtained from the Institutional Ethics Committee of Tatyasaheb Kore Dental College and Research Centre (TKDC/IEC/660/2025) prior to commencement, in adherence to the Declaration of Helsinki. All participants were provided informed consent through an online consent form presented at the start of the survey. The consent form detailed the study’s purpose, procedures, voluntary nature of participation, right to withdraw, and measures to ensure anonymity. Participation was voluntary, and no incentives were provided. The study involved 420 participants recruited from 16 dental colleges across India, with four colleges from each geographical zone (North, South, East, and West), using a secure online survey platform. The Institutional Ethics Committee of Tatyasaheb Kore Dental College and Research Centre provided central approval for the study, and written administrative permissions were obtained from all participating dental colleges for conducting the survey, ensuring compliance with institutional policies.

Sample size estimation

The sample size for this cross-sectional study was calculated using:

$$n = \frac{Z^{2} \times p \times (1 - p)}{d^{2}}$$

where Z = 1.96, p = expected proportion, and d = margin of error (0.05), resulting in a base sample of 352. Adjusting for a non-response rate of 15%, derived from a prior KAP survey on dentists in Maharashtra, India, which achieved an 84.94% response rate [10], the targeted distribution was increased to approximately 420 participants to maintain the statistical power for specialty-specific subgroups.

Eligibility

Eligibility for participation was restricted to full-time dental faculty members specializing in endodontics, prosthodontics, pedodontics, or public health dentistry, affiliated with accredited dental colleges in India, and possessing at least one year of teaching experience; exclusions were applied to part-time faculty, students, or individuals from unrelated specialties to focus on those actively involved in clinical education and practice.

Questionnaire development and validation

The questionnaire was prepared through a collaborative effort by a team of specialists, with Dr. Khushbu and Dr. Ketaki Rajguru (both endodontists) focusing on endodontic uses, Dr. Syed Aliya Haque (pedodontist) contributing pediatric perspectives, and Dr. Charu Khurana and Dr. Sumit Goyal (both public health dentists) addressing accessibility and public health angles. The questionnaire was developed through iterative meetings over two months, guided by a literature review and expert input. It comprises four sections: demographics (sex, specialty, teaching experience, and academic role), knowledge (four multiple-choice questions with each correct answer scored as 1 and incorrect answer scored as 0), attitudes (four questions recorded on a 5-point Likert scale: 1 = Strongly Disagree to 5 = Strongly Agree), and practices (four multiple-choice questions with each correct answer scored as 1 and incorrect answer scored as 0). The maximum score for knowledge and practice was 4, and the minimum was 0. For the attitude section, the minimum score was 4/4 = 1 and the maximum score was 20/4 = 5. The total KAP score ranged from 1 to 13 and was categorized as poor (≤50%), moderate (50-79%), and good (≥80%) [11] (see appendices). Validity was ensured through face validity (ten expert interviews with subsequent revisions) and content validity (scale-level CVI = 0.92). Reliability testing yielded Cronbach’s alpha values of 0.82-0.85 across the three domains.

A pilot study on 30 comparable faculty members showed no major issues and an average completion time of 15 min. Distribution occurred electronically via Google Forms, with links disseminated through professional emails, WhatsApp groups, and dental association networks using purposive sampling for balanced specialty representation. Collection spanned three months with bi-weekly reminders to non-responders, and responses were automatically aggregated into a secure spreadsheet for subsequent analysis.

Statistical analysis

Statistical analyses were performed by two statisticians (Dr. Pransi Gupta and Dr. Seema Gupta) using IBM SPSS Statistics for Windows, Version 26 (Released 2019; IBM Corp., Armonk, New York, United States), with data coded to ensure anonymity and facilitate analysis. The Kolmogorov-Smirnov test confirmed that the data were non-normally distributed. Consequently, the KAP scores were presented as medians and ranges. The Mann-Whitney U test was used to compare scores between sexes, and the Kruskal-Wallis test was applied for comparisons across specialties and designations, with the significance level set at p < 0.05. The relationships between the KAP scores and demographic variables were assessed using Spearman’s correlation coefficient. Additionally, a network analysis was conducted to evaluate the strength and influence of designation and specialty on the KAP scores.

## Results

In total, 370 (88%) participants responded, which included 197 (53.2%) female participants. Regarding specialty, the distribution was relatively balanced, with 105 (28.4%) endodontists being the most represented and 85 (23%) prosthodontists the least represented. By contrast, the distribution by academic role was highly skewed. The majority of individuals were readers, followed by professors, whereas senior lecturers constituted a clear minority of the academic sample. Based on the results of non-parametric tests, the total KAP scores differed significantly across key demographic variables. Male participants demonstrated a statistically higher mean KAP score (10.24 ± 0.87) than female participants (9.80 ± 1.10; p = 0.001). Significant disparities were also observed by specialty (p = 0.001), with endodontists scoring the highest (10.42 ± 0.92) and prosthodontists the lowest (9.57 ± 1.03). Furthermore, designation significantly influenced scores (p = 0.001); professors had the highest median knowledge and mean score (10.65 ± 0.82), while senior lecturers and readers demonstrated lower and comparable scores. These findings suggest that sex, professional specialization, and academic rank are important determinants of KAP in bioceramics and bioactive materials (Table 1).

**Table 1:** Comparison of knowledge, attitude and practice (KAP) scores between different categories of study variables. *p < 0.05 denotes statistical significance using the Mann-Whitney U test applied for the sex variable; **p < 0.05 denotes statistical significance using the Kruskal-Wallis test applied for specialists and designation variables. Categorical data are presented as N (%), where N represents the number of participants in each category, and % indicates the percentage of the total sample. Knowledge, attitudes, and practices (KAP) scores are reported as mean, median, and standard deviation (SD) to describe their central tendency and variability.

Variables	Category	N (%)	Median	Mean	Standard Deviation	Test statistics	p-value
Sex	Female	197 (53.2)	10	9.797	1.097	154.85	0.001*
	Male	173 (46.8)	10	10.237	0.867		
Specialists	Endodontists	105 (28.4)	10	10.424	0.918	39.34	0.012**
	Public health dentists	88 (23.8)	10	9.977	0.884		
	Pedodontists	92 (24.9)	10	10.13	1.04		
	Prosthodontists	85 (23.0)	10	9.571	1.027		
Designation	Senior lecturer	72 (19.5)	10	9.653	0.715	85.16	0.001**
	Professor	130 (35.1)	11	10.654	0.823		
	Reader	168 (45.4)	10	9.649	1.022		

The analysis revealed significant disparities in the KAP scores across demographic variables. Male participants exhibited significantly higher median good knowledge (p = 0.001), while female participants exhibited higher median good attitude (p = 0.001) scores. Specialization was a major determinant of knowledge and practice scores (p = 0.001), with endodontists and pedodontists consistently showing the highest median knowledge scores, while public health dentists showed good practice and attitude scores. However, no significant difference in attitude scores was found across specialties (p = 0.404). Academic designation significantly influenced all three domains (p = 0.001). Professors had the highest median knowledge and practice scores, yet readers and senior lecturers reported the highest median attitude scores. These findings imply that while sex and professional rank impact factual knowledge and clinical application, attitudes remain consistently positive across specialties (Table 2).

**Table 2:** Comparison of knowledge, attitude and practice (KAP) scores across demographic and professional categories. *p < 0.05 denotes statistical significance using the Mann-Whitney U test applied for the sex variable; **p < 0.05 denotes statistical significance using the Kruskal-Wallis test applied for specialists and designation variables. Knowledge, attitudes, and practices (KAP) scores are reported as median, and range.

Variables	Category	Knowledge score		Test statistics	p-value	Practice score		Test statistics	p-value	Attitude score		Test statistics	p-value
		Median	Range			Median	Range			Median	Range		
Sex	Female	3	2 - 4	11.48	0.016*	3	2 - 4	14.43	0.001*	4	2 - 4	19.57	0.001*
	Male	4	2 - 4			3	2 - 4			3	2 - 4		
Specialists	Endodontists	4	2 - 4	23.89	0.001**	4	2 - 4	33.28	0.001**	3	2 - 4	2.92	0.404
	Public health dentists	3	2 - 4			4	2 - 4			4	2 - 4		
	Pedodontists	4	2 - 4			3	2 - 4			3	2 - 4		
	Prosthodontists	3	2 - 4			3	2 - 4			3	2 - 4		
Designation	Senior lecturer	3	2 - 4	104.73	0.042**	3	2 - 4	91.73	0.001**	4	2 - 4	41.19	0.001**
	Professor	4	2 - 4			4	2 - 4			3	2 - 4		
	Reader	3	2 - 4			3	2 - 4			4	2 - 4		

A significant, strong positive correlation was found between experience and both knowledge (r = 0.565, p < 0.001) and practice (r = 0.312, p < 0.001) scores, indicating that greater experience was associated with higher knowledge and better practice. Similarly, higher academic designation (professor > reader > senior lecturer) showed strong positive correlations with knowledge (r = 0.529, p < 0.001) and practice (r = 0.37, p < 0.001). Conversely, attitude demonstrated significant weak negative correlations with both experience (r = -0.32, p < 0.001) and designation (r = -0.319, p < 0.001), suggesting that more senior and experienced faculty members held slightly less positive attitudes. Specialists showed weak positive correlations with knowledge and practice scores but no significant link to attitude scores (Table 3).

**Table 3:** Spearman correlation between knowledge, attitude and practice (KAP) scores and variables. Spearman’s rho represents the correlation coefficient between KAP scores and the variables experience, specialty, and designation, Specialty scores: Endodontists = 4, Pedodontists = 3, Public health dentists = 2, Prosthodontists = 1, Designation scores: Professor = 3, Reader = 2, Senior lecturer = 1. *p < 0.05 indicates statistical significance.

Variables		Experience	Specialists	Designation
Knowledge score	Spearman's rho	0.565	0.143	0.529
	p-value	0.001*	0.006*	0.001*
Practice score	Spearman's rho	0.312	0.291	0.37
	p-value	0.001*	0.001*	0.001*
Attitude score	Spearman's rho	-0.32	-0.005	-0.319
	p-value	0.001*	0.927	0.001*

**Figure 1:**
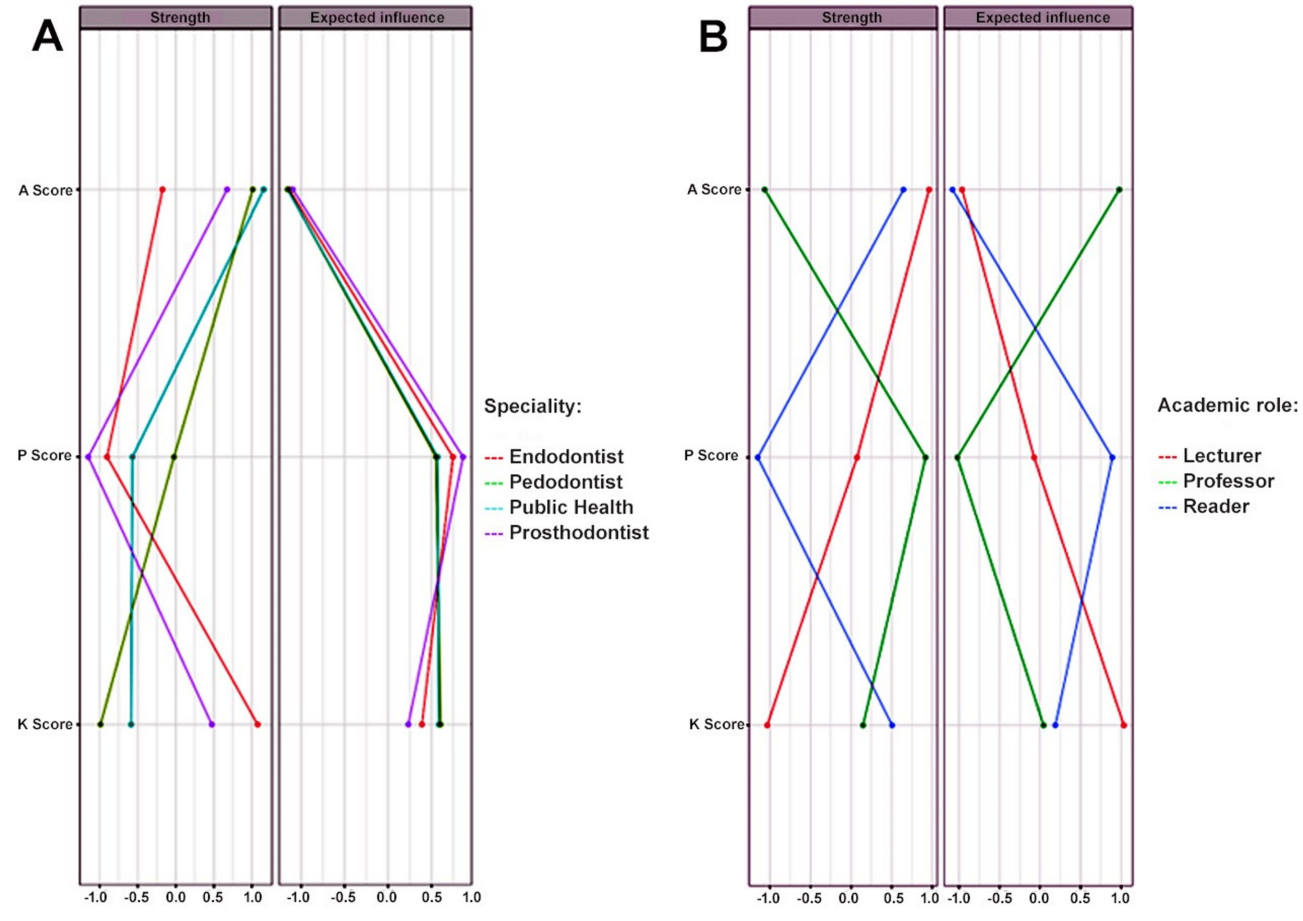
Network centrality plot of survey respondents. This plot illustrates the strength and expected influence centrality measures for participants within a professional network. Nodes represent individual respondents, colored according to their (A) specialty (Endodontist, Pedodontist, Public health dentist, Prosthodontist) and (B) academic role (Senior lecturer, Professor, Reader). The axes for both centrality measures are scaled from -1.0 to +1.0. This range indicates that connection weights within the network can be both negative and positive relationships. The plotted scores (KAP score) suggest the network analysis is based on a metric quantifying three dimensions (knowledge, practice and attitude) of the respondents.

The network analysis expected influence plot revealed a clear hierarchy based on the academic rank. Professors hold the highest positive centrality, identifying them as the most influential and activating hub within the network. Readers also demonstrated a positive, albeit moderately lower, level of influence. In contrast, senior lecturers exhibited a strong negative Expected Influence, functioning as an inhibitory node within the system. The results, supported by strong stability scores, suggested a significant disparity in network roles, with senior academic ranks acting as central drivers and senior lecturers occupying a peripheral or suppressive position (Figure 1).

The network analysis expected influence plot revealed a clear hierarchy among medical specialties. Endodontists emerged as the most central and positively influential hub, indicating a strong connection with other nodes in the network. Periodontists also showed positive, albeit lesser, influence. In stark contrast, prosthodontists functioned as strong inhibitory hubs with a significant negative influence, likely suppressing network activity. A neutral score for public health dentists suggests a peripheral role. These results, deemed highly stable by the correlation stability coefficient, suggest fundamentally different functional roles for each specialty within the system dynamics (Figure 1).

## Discussion

The observed disparities in the use of bioceramics and bioactive materials among dental academics underscore the multifaceted barriers to adopting innovation in clinical practice. These materials, prized for their biocompatibility and regenerative potential, represent a paradigm shift from traditional restoratives, yet their integration varies systematically according to individual and structural factors [1,8]. This variability not only reflects personal competencies, but also mirrors broader institutional dynamics, where professional hierarchies and experiential trajectories shape knowledge dissemination and behavioral change. 

The pronounced sex-based gradients in the cognitive and applicative domains challenge the prevailing narratives on sex in oral health behaviors. The results of our study indicated that while male participants showed good knowledge scores, female participants showed good attitude scores. This was likely due to the emphasis on technical skills in dental training, as supported by Lipsky et al. [12], who concluded that female participants show better attitudes than male participants about oral health literacy. Female participants had better knowledge scores, reflecting openness to new materials and dental health services, aligning with a previous study [13]. Sex-based training with hands-on and collaborative learning can balance these differences and improve the adoption of bioceramic and bioactive materials.

Our study found that dental specialty strongly affected knowledge and practice scores for bioceramics and bioactive materials but not attitude scores. Endodontists scored the highest, likely because they often use these materials, such as calcium silicate-based bioceramics [14]. These materials are central to their daily work, making them familiar with the benefits of bioceramics. Prosthodontists scored lower because their focus on fixed dentures rarely involves bioactive materials, reflecting training differences that prioritize mechanical solutions [15]. This pattern follows the diffusion of innovation theory, where specialists such as endodontists, who see clear benefits, adopt new materials faster [16]. However, all specialties showed similar positive attitudes, suggesting that enthusiasm exists but requires better training to boost knowledge and practice. Cross-specialty workshops could help to share expertise and promote the wider use of bioceramics and bioactive materials.

Our study found that more experienced dental academics and those with higher ranks, such as professors, had better knowledge and practice scores regarding bioceramics and bioactive materials. This is likely because professors often work on research projects, gaining deep understanding through repeated exposure to bioactive materials, such as those used in root canal treatments. According to a previous study, work experience is positively correlated with good knowledge and attitude scores [17]. Senior faculty with more than eight years of experience (professor level) help them master complex techniques, such as sealing with bioceramics, which aligns with adult learning ideas that practice improves skills. However, senior academics showed less positive attitudes toward bioceramics, possibly due to doubts from seeing issues such as imitations in solubility, dimensional stability (shrinkage and expansion), and retrievability [18]. This skepticism suggests the need for refresher training to boost enthusiasm and keep experienced professionals open to new developments.

Previous studies reported that the majority of participants were willing to use regenerative therapy, such as bioceramic and bioactive materials, mainly membranes and scaffolds, and felt that they should receive training to use them [19,20]. The majority were unaware of regenerative endodontic procedures using bioactive and bioceramic materials and their outcomes. This revealed that the residents were untrained in the use of sophisticated materials for regenerative endodontics. The increased cost associated with regenerative therapy constitutes the primary barrier to patient acceptance, as indicated by the majority of respondents [21]. The elevated costs of these therapies may stem from the utilization of more sophisticated equipment and the intensive labor required. However, with advancements in technology, there is potential for a substantial reduction in these expenses.

Our network analysis shows how influence spreads among dental academics. Professors act as key influencers by sharing knowledge about bioceramics quickly because of their central role, similar to how key researchers drive trends in medical education. Senior lecturers, nonetheless, possess diminished authority, which may constrain their contributions towards the enhancement of dental education. Within specialty networks, endodontists take the lead in advocating for bioceramics, whereas prosthodontists are generally more reserved. The positive dispositions evident across all factions reflect a keen interest in bioceramics, which can be harnessed through collaborative efforts to expedite their integration into dental practice, thereby mitigating the risks associated with regenerative treatment.

Notwithstanding the extensively recorded biological benefits associated with bioceramics and bioactive materials, their widespread utilization is hindered by deficiencies in training, knowledge, and systemic support. Tackling these impediments through focused educational programs and the integration of policy measures can improve the international application of these materials in regenerative procedures, thereby optimizing patient outcomes.

Limitations

This cross-sectional design, reliant on self-reports, invites response biases and limits generalizability. Confounding variables, such as institutional resources, were unaccounted for, and the absence of behavioral observables tempered causal inferences. Moreover, only faculty members were included in the survey, excluding undergraduate and postgraduate students. Therefore, prospective, high-quality, multicenter trials should be conducted to build clinical confidence in these materials, particularly in complex cases. Surveys should be conducted on a large scale incorporating undergraduates, postgraduates, and faculty members of different specialties. Qualitative interviews can explore senior lecturers’ attitudinal skepticism, inform targeted refreshers to boost enthusiasm, and ensure broader adoption of bioactive and bioceramic materials.

## Conclusions

This study demonstrated that KAP regarding bioceramics and bioactive materials were not uniform across dental faculty, but varied according to sex, specialty, and academic designation. Professors and endodontists emerged as the strongest advocates and influencers in their use, reflecting both experience and specialty relevance, while senior lecturers showed comparatively weaker engagement, highlighting an area for targeted support. Although knowledge and practice levels differed, the consistently positive attitudes across groups suggest that faculty members are receptive to adopting these materials, provided barriers of training, cost, and familiarity are addressed. The findings underscore the need for structured, cross-specialty training programs and faculty development initiatives that bridge gaps between specialties and ranks, ensuring that both technical knowledge and practical skills are strengthened. By leveraging the influence of senior faculty and fostering collaborative learning across specialties, the adoption of bioceramic and bioactive materials can be accelerated, ultimately enhancing the quality of restorative and regenerative dental care.

## Appendices

Tatyasaheb Kore Dental College and Research Centre

Department of Conservative Dentistry

Instructions

Anonymity: The survey was designed to be completely anonymous. No personally identifiable information (e.g., name, email address, employee ID) was collected at any point. The data collection platform (Google Forms) was configured not to capture IP addresses or email addresses.

Confidentiality: All collected data will be kept strictly confidential. It will be stored on a password-protected computer and/or encrypted cloud storage accessible only to the principal investigator and the immediate research team. The data will be used solely for the purposes of this academic research and will be aggregated for analysis, ensuring no individual's responses can be identified in any published or presented results.

Questionnaire

Part 1- Demographic Information

Age

Gender:

Specialty:

Years of Experience:

Academic Role:

Part 2- Participants' Response for KAP

Knowledge

**Table 4:** Questions for knowledge. This section tests your knowledge of bioceramic and bioactive materials. Please choose the single best answer for each question. Scoring: 1 point for each correct answer, total score = number of correct answers. Minimum score 0, maximum score 4.

Question no	Questions	Choices
1	The primary components of mineral trioxide aggregate (MTA) are:	a) Zinc oxide and Eugenol, b) Calcium silicates, c) Resin monomers and fillers, and d) Glass ionomers and polyacrylic acid
2	A key mechanism of action for bioactive restorative materials is:	a) Releasing antibiotics to kill bacteria, b) Physically blocking the dentinal tubules, c) Releasing calcium and phosphate ions to promote remineralization, and d) Creating a permanent adhesive seal with no ion exchange
3	Bioceramic materials are considered a gold standard for vital pulp therapy (e.g., direct pulp capping) because they:	a) Are highly cytotoxic and cause inflammation, b) Promote dentin bridge formation and have excellent biocompatibility, c) Have a low setting pH, and d) Are only indicated for non-vital procedures
4	Which material is commonly used for its osteostimulative properties in periodontal bone grafting?	a) Zinc phosphate cement, b) Gutta-percha, c) Bioactive glass (e.g., NovaBone, PerioGlas), and d) Resin-Modified GIC

Attitudes

**Table 5:** Questions for attitude. Please indicate your level of agreement with the following statements. Scale: 1 = Strongly Disagree, 2 = Disagree, 3 = Neutral, 4 = Agree, 5 = Strongly Agree. Minimum average score = 1, maximum average score = 5.

Question no	Current Question	Scale of agreement
5	Bioceramic and bioactive materials should be integrated more widely into dental curricula to prepare future practitioners for minimally invasive techniques.	Strongly disagree/Disagree/Neutral Agree/Strongly agree
6	The higher cost of bioceramic materials outweighs their clinical benefits in routine restorative procedures.	Strongly disagree/Disagree/Neutral Agree/Strongly agree
7	I believe that bioactive materials represent a significant advancement over traditional composites in promoting long-term tooth preservation.	Strongly disagree/Disagree/Neutral Agree/Strongly agree
8	I find the handling properties of some bioceramic materials (e.g., setting time, consistency) to be a significant barrier to my use.	Strongly disagree/Disagree/Neutral Agree/Strongly agree

Practice

**Table 6:** Questions for practice. Please review the following clinical scenarios and select your most likely treatment choice based on current evidence and your clinical judgment. Scoring: Score based on the evidence-based appropriateness of the choice (e.g., 1 point for selecting the most appropriate bioceramic/bioactive option). Minimum score 0, maximum score 4.

Question no	Current Question	Choices
9	Scenario: A 22-year-old patient presents with a deep, carious exposure on a vital, asymptomatic first molar. Pulp vitality tests are normal. Your preferred material for direct pulp capping is:	a) Calcium hydroxide, b) Bioceramic cement (e.g., MTA, Biodentine), c) Zinc Oxide Eugenol (ZOE), and d) Glass Ionomer Cement
10	Scenario: You are restoring a class II cavity on a high-caries-risk patient. You are concerned about preventing recurrent caries around the margins. Your material of choice is:	a) Amalgam, b) Conventional Composite Resin, c) Bioactive restorative (e.g., Glass Hybrid, Giomer, or Cention N), and d) Resin-Modified GIC
11	Scenario: A 45-year-old patient with a history of periodontitis presents with a deep, narrow, 3-wall intra-bony defect on a mandibular molar. The tooth is vital and has good prognosis. Your preferred material for achieving predictable bone regeneration is:	a) Autogenous bone graft alone, b) A synthetic bone graft containing bioactive glass (e.g., NovaBone, PerioGlas), c) Demineralized Freeze-Dried Bone Allograft (DFDBA), and d) No grafting, perform open flap debridement only
12	Scenario: During non-surgical retreatment of a previously root-filled maxillary central incisor, you encounter a perforation in the furcation area. The perforation is small (<2mm) and not contaminated. Your preferred material for sealing the perforation is:	a) Composite Resin, b) Bioceramic cement (e.g., MTA, Biodentine), c) Glass Ionomer Cement, and d) Zinc Oxide Eugenol (ZOE)
